# BioNetBuilder2.0: bringing systems biology to chicken and other model organisms

**DOI:** 10.1186/1471-2164-10-S2-S6

**Published:** 2009-07-14

**Authors:** Jay H Konieczka, Kevin Drew, Alex Pine, Kevin Belasco, Sean Davey, Tatiana A Yatskievych, Richard Bonneau, Parker B Antin

**Affiliations:** 1 Department of Molecular & Cellular Biology, University of Arizona, Tucson, AZ, USA; 2 Courant Institute, New York University, New York, NY, USA; 3 Department of Biology, New York University, New York, NY, USA; 4 Department of Cell Biology & Anatomy, University of Arizona, Tucson, AZ, USA

## Abstract

**Background:**

Systems Biology research tools, such as Cytoscape, have greatly extended the reach of genomic research. By providing platforms to integrate data with molecular interaction networks, researchers can more rapidly begin interpretation of large data sets collected for a system of interest. BioNetBuilder is an open-source client-server Cytoscape plugin that automatically integrates molecular interactions from all major public interaction databases and serves them directly to the user's Cytoscape environment. Until recently however, chicken and other eukaryotic model systems had little interaction data available.

**Results:**

Version 2.0 of BioNetBuilder includes a redesigned synonyms resolution engine that enables transfer and integration of interactions across species; this engine translates between alternate gene names as well as between orthologs in multiple species. Additionally, BioNetBuilder is now implemented to be part of the Gaggle, thereby allowing seamless communication of interaction data to any software implementing the widely used Gaggle software. Using BioNetBuilder, we constructed a chicken interactome possessing 72,000 interactions among 8,140 genes directly in the Cytoscape environment. In this paper, we present a tutorial on how to do so and analysis of a specific use case.

**Conclusion:**

BioNetBuilder 2.0 provides numerous user-friendly systems biology tools that were otherwise inaccessible to researchers in chicken genomics, as well as other model systems. We provide a detailed tutorial spanning all required steps in the analysis. BioNetBuilder 2.0, the tools for maintaining its data bases, standard operating procedures for creating local copies of its back-end data bases, as well as all of the Gaggle and Cytoscape codes required, are open-source and freely available at http://err.bio.nyu.edu/cytoscape/bionetbuilder/.

## Background

Molecular interaction databases have made searching for interactions between interesting genes easier, and have thus brought countless new hypotheses to the work of researchers around the globe. Standardized data exchange formats such as PSI-MI [1] and BioPAX [2] have facilitated communication of large amounts of data, stretching the reach of genome- and proteome-wide interaction data in biological research. The usefulness of these data to researchers has been dramatically enhanced by Cytoscape [3], the popular network visualization and analysis software, which provides a platform to integrate and visualize data in the context of molecular interaction networks [4]. Although data exchange formats are widely supported among interaction databases, not all represent interactions in the same way, particularly with respect to the identifiers used to represent the genes. BioNetBuilder [5] is an open-source client-server Cytoscape plugin that addresses this issue by integrating molecular interaction databases, the Gene Ontology, and Gaggle-enabled tools to generate and serve whole or partial networks to a user's Cytoscape environment.

In version 2.0 of BioNetBuilder, we have made significant improvements to expand the volume and usability of interaction data for Cytoscape users. To begin, we added the IntAct [6], MINT [7], and MPPI [8] interaction resources. The list of integrated databases now includes BIND [9], BioGrid [10], DIP [11], HPRD [12], IntAct, KEGG [13], MINT, MPPI, and Prolinks [14]. The integration of these networks is made possible via a new synonyms-resolution system that provides a means of translating between the many different identifiers used by each individual database or tool. The synonym translator provides mappings for gene/protein identifiers based largely on the RefSeq [15] and iProClass [16] databases. While the previous version of BioNetBuilder allowed users to assign a variety of alternate gene/protein names as node attributes in Cytoscape, networks could only be constructed with Refseq protein GI numbers as the Cytoscape node identifiers. This was limiting because present versions of Cytoscape do not allow users to exchange node identifiers for node attributes, and many downstream analysis tools operate only on node identifiers. The second major improvement in version 2.0 is that BioNetBuilder now allows users to select from a diverse list of id types to set as the Cytoscape node identifiers; this greatly increases the interoperability of our tools with other downstream analysis (such as Gaggle-enabled tools and other Cytoscape plugins). This interoperability was a key prerequisite to our third main improvement, which is that BioNetBuilder 2.0 is now Gaggle enabled [17].

The Gaggle is a cross-platform data integration system designed to allow seamless shuttling of biological data across applications. BioNetBuilder 2.0 can construct interaction networks based upon data received from any resource with a plugin that implements the Gaggle framework, including but not limited to: Cytoscape itself; MeV, a data matrix viewer for navigating and plotting high throughput experimental data; R, the open-source statistical computing software; MatLab, the scientific computing software; and the web browser Firefox, which enables communication with KEGG, STRING [18], and DAVID [19]. Additionally, information from networks constructed with BioNetBuilder can be used to transfer data to any of the aforementioned resources using the Cytoscape Gaggle plugin CyGoose.

The fourth and most significant improvement to BioNetBuilder 2.0 is that interactions can be transferred across species. Although more than 500 species are represented in BIND, BioGrid, DIP, HPRD, IntAct, MINT, and MPPI, most model organisms, including chicken, have relatively little interaction data available. To address this problem, all interactions in the databases listed above were used to compute integrated interolog networks for many eukaryotic model systems, including chicken [20]. An interolog, or interaction homolog, of a pair of interacting proteins (A, B) can be faithfully transferred from one species to another if the orthologs to (A, B): (A', B'), share at least 80% joint sequence identity to (A, B) [21]. As reported by Konieczka et al. [20], coverage of the chicken interactome improved dramatically when interolog-derived interactions were included.

To facilitate the use of BioNetBuilder 2.0 and Cytoscape by researchers who may have little formal bioinformatics experience, we provide a tutorial on how to use BioNetBuilder 2.0 to download and use a custom chicken interactome. In the first section, we describe step-by-step construction of a customizable interactome for chicken. In the second section, we show how to integrate a sample microarray data set into the network and apply a method for extracting relevant subnetworks. Finally we discuss how to use information in a subnetwork to generate novel biological hypotheses.

## Methods

### BioNetBuilder 2.0

BioNetBuilder consists of a client, explained further below, and a Java servlet as explained in greater detail by Avila-Campillo et al. [5]. A major change in the version 2.0 client and servlet came as a result of an upgrade of the Apache XML-RPC to the latest version (3.1). This paved the way for critical changes to the way interactions are passed across the web.

Another major change in version 2.0 lies in how interactions are stored. In the original version of BioNetBuilder, design was centered on flexibility for developers to add new interaction resources. In version 2.0, we have added the constraint that the internal node identifiers for any interaction resource be RefSeq protein GI numbers. This is because our Synonyms Resolution engine, largely powered by the iProClass database, refers every id type translation through RefSeq protein GIs. Although this has the disadvantage of losing interactions from some databases, we have found these losses to be relatively negligible. Advantages however, include significant improvements in the speed of database access, as well as several advantages for new developers trying to understand the BioNetBuilder code required to add custom databases. BioNetBuilder 2.0 also allows client-side users to select the id types to be returned to Cytoscape (or any other tool querying the BioNetBuilder back-end database).

The Synonyms Resolution engine (now centered on RefSeq protein GIs) can also be used to translate interactions across species via the Interologger. This engine incorporates a local installation of the NCBI database HomoloGene and recently the orthology database InParanoid [22]. Interologs are computed and integrated as described in Konieczka et al. [20], and stored in the BioNetBuilder 2.0 database so they can be served to the client seamlessly along with other data services.

### Microarray data

Epiblast, primitive streak, and mesoderm cell layers from Hamburger-Hamilton stage 4 white leghorn chicken embryos [23] were microdissected and pooled into 3 respective tissue samples. RNA was extracted, amplified, labelled and hybridized according to standard kits and protocols by the Genomics Research Laboratory at the University of Arizona. Hybridizations were performed using a custom 20,460-feature long-oligo microarray. A standard wheel design was used for comparing three samples, in which each sample is compared to the other, with a dye swap for each comparison, for a total of six microarray chips. To normalize the results, a custom pipeline was written in the R statistical computing language. Within-chip normalization was performed using the R package OLIN (Optimized Location- and Intensity-dependent Normalization) [24]. The False Discovery Rates were then computed for each spot based on intensity- and location-dependent bias. Spots demonstrating a bias were removed from downstream analysis. Having normalized spots within chips, standard libraries in the R BioConductor package were used to normalize between chips [25]. Finally, linear models were fit to the normalized gene expression data using the limma library, which computes log_2 _fold-change, indicating the direction and quantity of the differential gene expression between the samples, summary statistics including T- and B-statistics, and the adjusted p-value that takes into account the false discovery rate [26].

## Results and discussion

**Figure 1:**
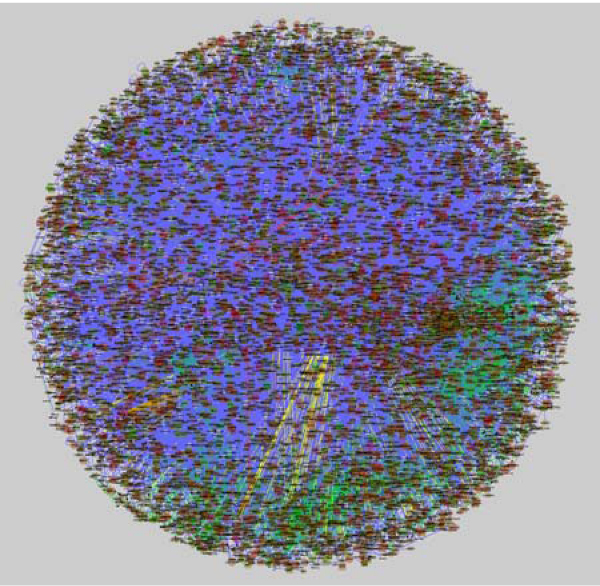
**Chicken interactome**. View of the chicken interactome with 72,000 interactions among 8,140 genes downloaded in Cytoscape with BioNetBuilder 2.0, showing the density of data contained within a network of this size.

To illustrate the utility of BioNetBuilder 2.0 to researchers working with avian models, we will demonstrate how it can be used to create a chicken interactome containing 72,000 interactions among 8,140 genes, with interactions mapped from all sources listed above (Figure 1). To demonstrate one of many possible interoperative tools, the following tutorial takes the user step-by-step through the process of construction, use, and analysis of this interactome using the jActive Modules Cytoscape plugin. This example uses all interactions available for chicken and, depending on the computer, the entire tutorial can take several hours to two days to complete. We therefore also provide precomputed files for download to allow users to skip the most time consuming steps. We also illustrate an alternate use-case in the supplemental material, using a subset of interaction data that demonstrates another way in which BioNetBuilder can be used without the overhead of constructing and analyzing the entire interactome.

### Building a custom chicken interactome with BioNetBuilder 2.0

Before getting started, download and install Cytoscape 2.6 from http://www.cytoscape.org. We encourage new users to go through the Cytoscape basic online materials http://cytoscape.org/cgi-bin/moin.cgi/Presentations/Basic, which are well designed to acclimate users to the Cytoscape environment and its basic functions. You will also need data from the supplemental data package for this tutorial: BioNetBuilderData.tgz (Additional file 1). Unpack the file by double clicking it, and a directory called "BioNetBuilderData" will be created. Refer to this for files needed throughout the tutorial.

Next, launch Cytoscape 2.6 and install the BioNetBuilder2.0 plugin and a plugin called jActiveModules [27]. This can be accomplished manually or through the Cytoscape plugin manager. To load plugins through the plugin manager, click the ***Plugin ***drop-down menu and select ***Manage Plugins***. This will open the "Manage Plugins" window, showing a list of plugin types available for installation. Click the arrow next to the ***Analysis ***folder to open it, select ***jActiveModules***, and click ***Install***. Then click the arrow next to the ***Network and Attribute I/O ***folder to open it, select ***BioNetBuilder2.0***, and click ***Install***. To install BioNetBuilder 2.0 manually, download the plugin from http://err.bio.nyu.edu/cytoscape/bionetbuilder/downloads.php and place the bionetbuilderClient.jar file in the "plugins" directory found inside your Cytoscape application folder.

Restart Cytoscape, and you are ready to begin the tutorial for building a custom chicken interactome:

1. Under the ***Plugin ***drop-down menu, you should now have an option to select the ***BioNetBuilder Wizard***... button to launch BioNetBuilder. Type "gallus" in the "Species search string:" text box and click ***Search***. The filename "SpeciesName=Gallus gallus, TAXID = 9031" will be returned in the text area below; select that line in the text area (Additional file 2 Figure S1a) and click ***Next ***>.

2. The Node ID type selection pane should now be visible with five ordered options for selecting Node ID types. These are used for selecting your preference for node identifiers. These preferences are used to create network nodes in decreasing order of priority. If all nodes are available in the first priority you set, these are the only id types that will be present in your network. If a node cannot be mapped to this id type, however, BioNetBuilder attempts to map the node to your second preference, and so on. If none of your priorities can be found, the node will not be created. As mentioned above, the default identifier type for nodes in BioNetBuilder is Refseq Protein GI numbers (GI). To guarantee return of all nodes and interactions available, set "GI" as the first priority ID type. However, for this tutorial, we will need to have all of our node identifiers as Entrez Gene UID numbers. Select **GeneID **for **each of the 5 options **(Additional file 2 Figure S1B), then click ***Next ***>. This guarantees that all nodes in the network will have Entrez GeneIDs as identifiers since this is the only option available. If a node does not map to an Entrez GeneID, it will not be included in the network.

3. You will now see a pane for selection of Node Sources for your Biological Network (Additional File 2 Figure S1C). We will not use this for the main tutorial, so you can click ***Next ***> and go to step 4. Alternatively, at this point proceed to step 3 in the supplemental tutorial to avoid construction of the entire interactome. In the supplement, we demonstrate construction of a smaller network from a set of starting nodes, which can be any combination of the following: nodes with certain GO annotations, nodes from a custom starting list (as in the supplemental tutorial), nodes found by searching names at NCBI, nodes from another Gaggle resource, or nodes from another network loaded into Cytoscape. You would click on the check box to the left of the resource to make the button available, then click the button itself for further options.

4. You will now see the pane for selecting "Data Sources for the Edges in your Network". Click the boxes next to the following to select the resources that could have interactions available for chicken: ***BIND***, ***BioGrid***, ***DIP***, ***IntAct***, ***Interologger***, ***KEGG***, ***MINT***, and ***MPPI***. Now click ***Calculate number of edges from selected databases ***to display the number of interactions available for chicken from each resource (Additional File 2 Figure S1D). Because few interactions have been verified directly in chicken, almost all of the interactions come from KEGG and Interologger, the latter being those transferred from other species. If you wish to restrict this set based on scores and/or interaction types, you may do so by clicking on the ***Interologger***... button and setting your criteria. The confidence threshold is a value (between 0 and 1) above which interolog-derived interactions will be included. The scoring system is explained in detail in Konieczka et al [20]. For this tutorial, leave the threshold set at 0.0 in order to return all available interactions. Click ***Next ***> to advance to the attributes pane.

5. You should now see the pane for selecting "Attributes You Wish to Add to Your Network" (Additional File 2 Figure S1E). Although all node identifiers will be Entrez Gene UIDs due to our selection in step 2, we can attach various other data to our nodes as well by selecting them here. When you are satisfied with your selections, click ***Next ***> to advance to the Network name panel.

6. Finally, set your "Network Name" by entering it in the text field (Additional file 2 Figure S1F). Click ***Finish ***to construct your network, which is very large and depending on the speed of your computer and internet connection can take from minutes to hours to construct.

If you do not wish to download the network through BioNetBuilder, the network is included as a file in the supplemental data package called "chicknet.xgmml", which can be imported into Cytoscape by selecting ***File ***→ ***Import ***→ ***Network (multiple file types).... ***Once your network is finished downloading, or importing, a view will not be created due to its size. You can see the summary information for the network by clicking the ***Network ***button in the "Control Panel" on the left side of the Cytoscape window. It may be necessary to scroll to find the ***Network ***button using the left arrow in the "Control Panel (Additional file 2 Figure S1G-H). We do not need to view the network at this point. However, if desired, you can create a view by right-clicking on the network name in the "Control Panel" and selecting ***Create View***, but this is not recommended at this point since it will require a great deal of memory to render such a large network. At time of publication, this particular configuration loaded a network with 72,000 edges connecting 8,140 nodes. To convey the density of information within it, a view of the rendered network is shown in Figure 1.

### Getting value from the chicken interactome

In this section of the tutorial, we will take you through the process of integrating an expression data set with your newly constructed interactome, and then extracting a relevant subnetwork. For this example, we will use expression data from experiments comparing gene expression differences as cells undergo a transition from the epithelial epiblast to form the primary germ layers during gastrulation. This is a fundamental transformation that occurs during early development of most multicellular organisms and is regulated through numerous evolutionarily conserved pathways. The question we are addressing is, "what are the core modules of genes driving the progression of cells from the epiblast through the primitive streak to become mesoderm during chicken gastrulation?" As will be made clearer below, this will set the stage for more specific questions once we have completed this first phase of analysis. To address this question we will use statistical results from two microarray comparisons: primitive streak vs. epiblast, and mesoderm vs. primitive streak. Both comparisons are derived from the same microarray study using pooled tissue dissections of epiblast, primitive streak, and newly formed mesoderm from HH stage 4 chick embryos [23]. The statistics provided result from analysis of a standard 3-way wheel design with 6, 2-color hybridizations on a custom 20,460-feature long-oligo microarray.

Complete the following steps to integrate the expression data and extract a subnetwork that addresses the fore-mentioned question:

1. Find the files "streak_vs_epiblast.pvals" and "mesoderm_vs_streak.pvals" in the supplemental data package. These files contain mappings from Entrez Gene IDs (first column) to expression values (log_2 _fold changes listed in the second column) and a statistic estimating the probability of differential expression (adjusted p-values listed in the third column).

2. Import each by clicking ***File ***→ ***Import ***→ ***Attribute/Expression Matrix... ***Leave the assignment of nodes set to ID and click ***Import***. Repeat this process for the second file. The expression data are now attached to the nodes as attributes and can be viewed by first selecting all nodes (***Select ***→ ***Nodes ***→ ***Select all nodes***); then selecting "streak_vs_epi_exp", "streak_vs_epi_sig", "meso_vs_streak_exp", and "meso_vs_streak_sig" from the "Select Attributes" menu in the "Node Attribute Browser". This browser is visualized in the Data Panel at the bottom of your Cytoscape window by clicking ***Node Attribute Browser ***(Additional File 3 Figure S2A-C).

3. If you are following the alternate tutorial (Additional file 4 and 5) provided in the supplement, or if you would prefer not to spend the time required, you may want to skip these last two steps. Depending on the speed of your computer, this program can run for a few hours or even overnight. Launch jActiveModules by selecting it from the ***Plugins ***menu. ***jActiveModules ***will appear in the "Control Panel" on the left side of your Cytoscape menu. Select "streak_vs_epi_sig" and "meso_vs_streak_sig" by clicking once on each in the "Expression Attributes for Analysis" text area (Additional File 3 Figure S2D). Leave the default settings and click ***Find Modules ***at the bottom of the panel.

4. Once jActiveModules finishes computing, a "Results Panel" will appear on the right side of your Cytoscape window (Additional File 3 Figure S2E). Here you will find five subnetworks each with scores indicating the relative strength of the respective subnetwork. To create a network from any of these subnetworks, select the desired subnetwork by clicking directly on the number, then click ***Create Network ***at the bottom of the "Results Panel".

**Figure 2:**
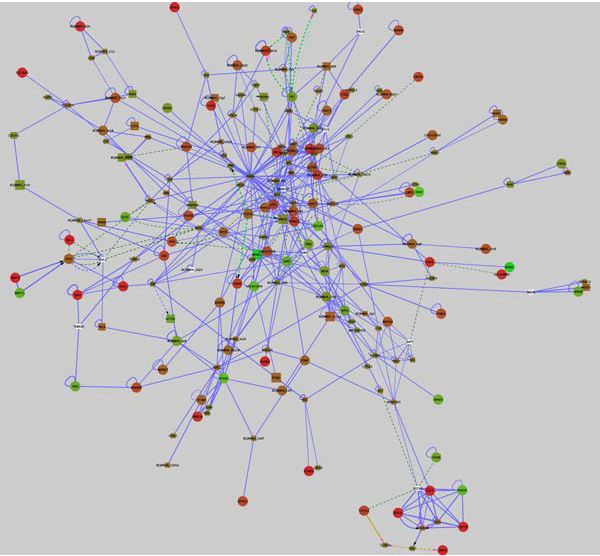
**Epiblast-streak-mesoderm subnetwork**. A subnetwork of the chicken interactome returned from jActiveModules using microarray data representing the progression from the epiblast through the streak to become developing mesoderm. This subnetwork represents a co regulated unit containing196 nodes and 553 edges. Nodes are colored according to gene expression: green represents down-regulation in the primitive streak relative to the epiblast, red represents up-regulation, and white indicates a lack of expression data. Node size indicates significance of differential expression, whereby genes with lower adjusted p-values are larger. Circular nodes are nodes meeting the threshold for significance, adj. p ≤ .08, whereas rectangular nodes either have adj. p-values greater than this value or do not have expression data associated. Interactions are color-coded according to interaction type: blue = protein-protein, yellow = gene expression, dark green = genetic interaction, and light green = part of enzymatic compound. Interactions that are direct are solid lines, whereas dashed lines indicate indirect interactions.

Although often similar, the subnetworks returned by jActiveModules are not identical, so we have provided a network we computed in exactly the way described above in the supplemental data package called "ESM_subnetwork.xgmml". Import this network by clicking ***File ***→ ***Import ***→ ***Network (multiple file types)... ***An important feature of Cytoscape is the ability to visualize networks in various customizable styles through the VizMapper™. We have also provided Vizmap property files in the supplemental data package: "streak_vs_epiblast.props" and "mesoderm_vs_streak.props", either of which can be loaded by clicking ***File ***→ ***Import ***→ ***Vizmap Property File... ***Once the visual style is loaded, it can be applied to the network by selecting ***VizMapper***™ in the "Control Panel" on the left side of the Cytoscape window, then selecting the visual style from the drop-down menu (Additional File 3 Figure S2F). Instruction for creating and modifying visual styles can be found in the Cytoscape online tutorials mentioned above. After loading the subnetwork we provided and importing and selecting the "streak_vs_epiblast" visual style, your Cytoscape window should look exactly like the network shown in Figure 2. The individual gene expression profiles can be selected to view as attributes in the ***Node Attribute Browser ***of the "Data Panel" at the bottom of your Cytoscape window as described above in step 2 of the second tutorial. To visualize the mesoderm vs. streak gene expression differences, load the "mesoderm_vs_streak.props" Vizmap property file and select it as described above. To search for specific gene names in the network, click the "Configure search options" button to the right of the "Search:" menu (Additional file 3 Figure S2G), and select the "GeneName" attribute from the list of Node options (Additional file 3 Figure S2H).

The network in Figure 2 represents a subnetwork of the chicken interactome that is highly differentially expressed, relative to other subnetworks, in the progression of cells from the epiblast through the primitive streak to become mesoderm. The implication is that within this subnetwork are smaller modules, or at least interacting components, necessary to drive cells through this progression.

A major strength of visualizing gene expression data layered onto interaction networks is that the networks contain large numbers of molecular relationships, only some of which might be discoverable from the published literature. Novel relationships between groups of co-regulated molecules can be rapidly identified, frequently linked through molecules that are not differentially expressed but nevertheless are important components of the overall functional network. This latter category of molecules is not readily identifiable through standard analysis of microarray data.

To illustrate how subnetworks derived from layering expression data onto the chicken interactome can illuminate biological processes, we will focus on two small portions of the subnetwork shown in Figure 2. Here we are examining changes in gene expression between cells of the epiblast, an epithelium that surrounds the primitive streak, and cells of the primitive streak region, where many cells are undergoing changes associated with the epithelial to mesenchymal transition (EMT) that leads to the formation of endoderm and mesoderm. Cells undergoing EMT in the primitive streak are derived from the more lateral epiblast. This process involves large-scale changes in transcription mediated by cell signalling pathways, and changes in cell shape and movement.

**Figure 3:**
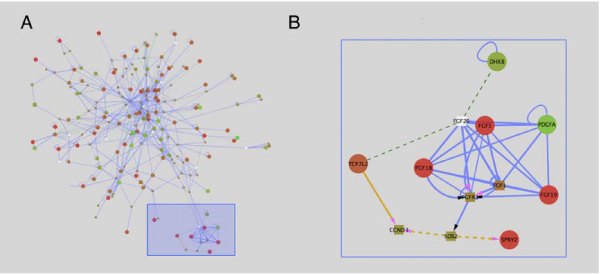
**Fgf Subnetwork**. (A) A module of the subnetwork in Figure 2 is highlighted by the blue box. This module is enlarged in (B), and contains several FGF ligands whose expression is highly upregulated in the primitive streak relative to the epiblast. An important aspect of these modules is that molecules showing changes in gene expression (red or green circles) are linked through molecules whose expression does not change. FGF signalling is known to be active in the primitive streak region relative to the more lateral epiblast.

First we examine a small module of the subnetwork that validates existing knowledge concerning mechanisms regulating gastrulation. The first subnetwork (Figure 3) shows a collection of linked nodes that represent several Fgf ligands, including FGFs 1, 3, 18, 19, 20 and FGF receptor 3 (FGFR3). Of these, Fgfs 3, 18 and 19 are upregulated, indicating that FGF signalling is activated in the primitive streak region relative to the more lateral epiblast. SPRY2, a negative modulator of Fgf signalling that is typically expressed in regions of active Fgf signalling, is also upregulated. In contrast, PDGFA, which is known to interact with FGF ligands, is downregulated in the primitive streak relative to the epiblast. Fgf signalling is a known regulator of the transcriptional and cell migration changes that occur during gastrulation [28,29], and as such, it is reassuring to see these signalling molecules present as a module in this subnetwork.

**Figure 4:**
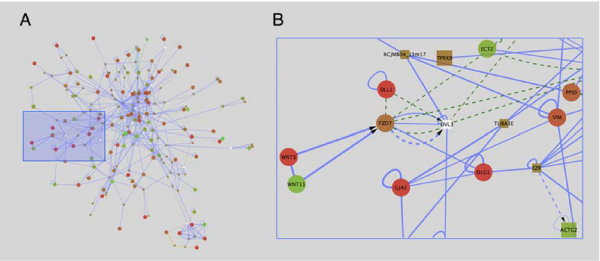
**Wnt subnetwork**. (A) A second module of the subnetwork from Figure 2 is I highlighted by the blue box, and is enlarged in (B). This module links Wnt signalling through WNT3 and WNT11 ligands and the WNT receptor FZD7, to several cytoskeletal molecules including DLG1 (discs large), GJA1 (connexin43) EZR (ezrin), VIM (vimentin) and TUBA3E (tubulin).

The second module (Figure 4) encompasses nodes and edges that link several cytoskeletal proteins associated with cell junctions in epithelial cells to the Wnt signalling pathway. Wnt intercellular signalling molecules such as WNT3 and WNT11 bind to frizzled cell surface receptors (FZD7), and mediate downstream signalling. While "canonical Wnt signaling" leads to changes in gene transcription, the so-called "non-canonical Wnt signalling" pathways regulate cell shape and movement [32,33]. In chicken embryos it has recently been shown precise levels of signalling through the non-canonical pathway mediated by the WNT11 homologue WNT11B, regulates migration of cells from the epiblast through the primitive streak during gastrulation [30].

Of particular interest in this module is the linkage between FZD7 and DVL3 (Dishevelled), and several cell junction associated proteins including DLG1 (discs large), GJA1 (connexin 43), EZR (ezrin), and two tubulin proteins (TUBA3E and TUBA3C). In Drosophila, DLG1 is required for maintenance of certain junctional complexes, and lack of this protein leads to loss of many aspects of epithelial polarity and disruption of the microtubule and actin cytoskeleton [31]. While the primitive streak is a region in which cells are losing epithelial characteristics, our microarray studies show that DLG1 expression is strongly upregulated in the primitive streak region relative to the epithelial epiblast. Although published data implicates non-canonical Wnt signalling as a positive regulator of cell migration [30,32,33], DLG1 has been broadly implicated in maintaining integrity of the epithelium, aberrations of which are responsible for certain human diseases [34,35]. These findings suggest that one or more negative modulators of EMT, including DLG1, are activated in the primitive streak. The relationship between non-canonical Wnt signalling and DLG1 expression and function within the primitive streak remains conjectural until further explored experimentally. Nevertheless, combining the chicken interactome with expression data has uncovered an intriguing and potentially significant group of biological interactions would have otherwise remained unrecognized.

## Conclusion

BioNetBuilder 2.0 introduces a new suite of tools to the chicken model system, and by extension many other model systems [20], that are user-friendly and interoperable with a wide variety of other useful programs via an easy to use (and already widely used) interface. By transferring and integrating interactions across species and serving them directly to Cytoscape, with any combination of previously available interaction data, a very large chicken interaction network is available for experimental investigation. Users can take advantage of the numerous plugins for downstream analysis as well as all resources available through the Gaggle.

## Competing interests

The authors declare that they have no competing interests.

## Authors' contributions

JHK, KD, AP, KB, and SD implemented the improvements to BioNetBuilder. JHK wrote the manuscript, generated the figures, and performed statistical analysis of the microarray data. TAY planned and performed the chicken embryo dissections for microarray analysis. RB supervised aspects of BioNetBuilder 2.0 development and wrote and edited portions of the manuscript. PBA designed and coordinated the studies, interpreted data and wrote the manuscript. All authors read and approved the manuscript.

## Supplementary Material

Additional file 1 This file contains all necessary data in the tutorial. It can be unpacked by double-clicking it. Click here for file

Additional file 2 This file contains screen-shots of each step of part 1 of the main tutorial to help user's follow along. Click here for file

Additional file 3 This file contains screen-shots of each step of part 2 of the main tutorial to help user's follow along. Click here for file

Additional file 4 Alternate tutorial. Click here for file

Additional file 5 This file contains screen-shots of each step of the alternate tutorial to help user's follow along. Click here for file
