# Supplementary figures and images for: BioNetBuilder2.0: bringing systems biology to chicken and other model organisms
Source: BMC Genomics. 2009 Jul 14;10(Suppl 2):S6. doi: 10.1186/1471-2164-10-S2-S6 (PMC2966329; doi:10.1186/1471-2164-10-S2-S6)

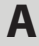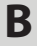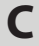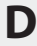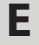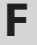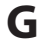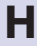

Supplement: Additional file 2 — This file contains screen-shots of each step of part 1 of the main tutorial to help user's follow along. [file 1471-2164-10-S2-S6-S2.pdf]

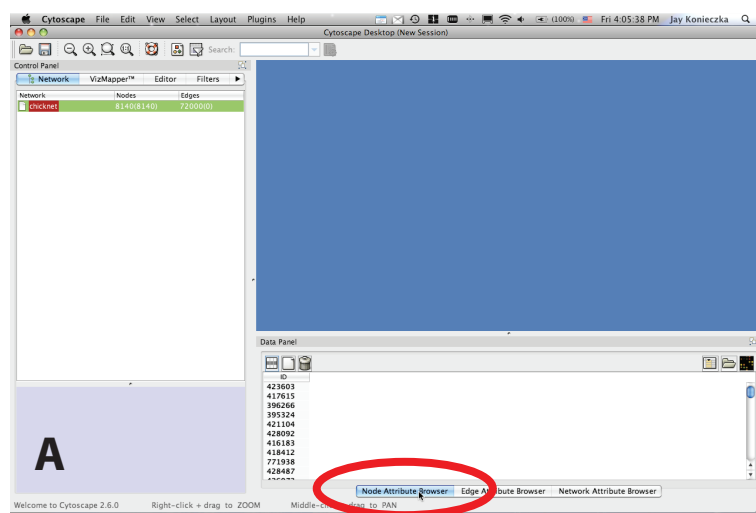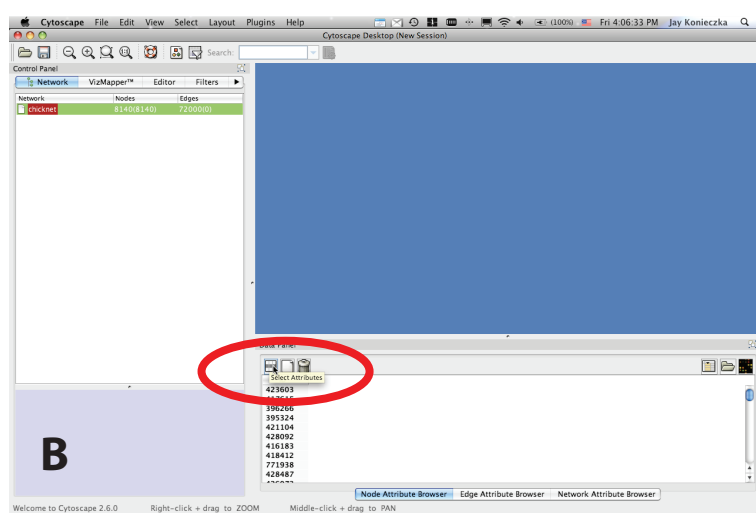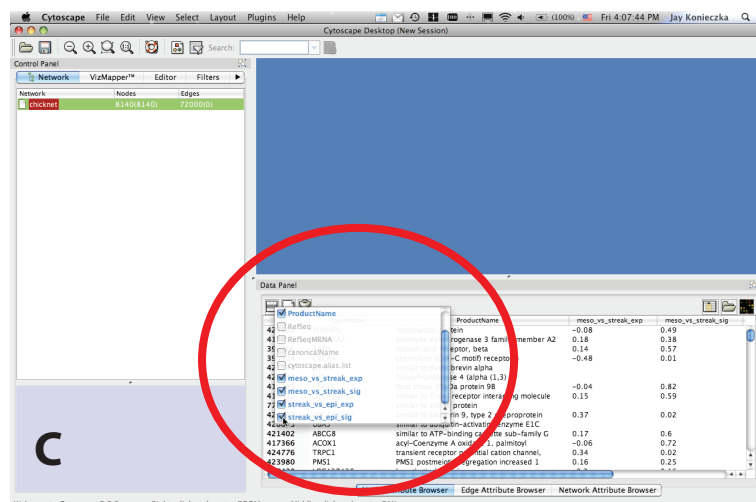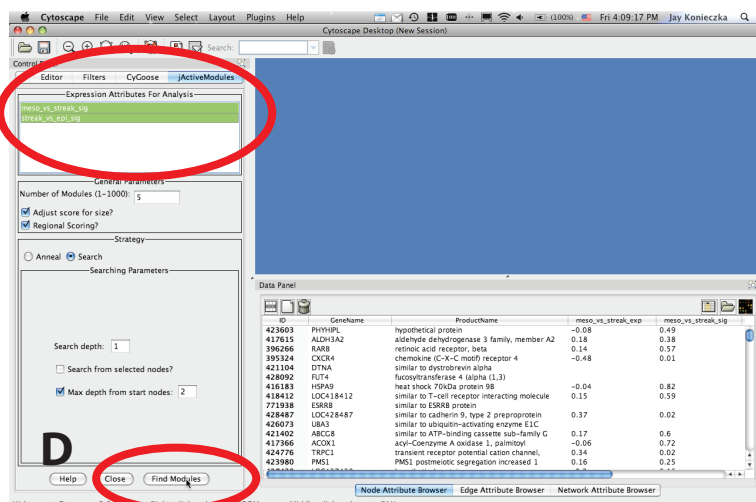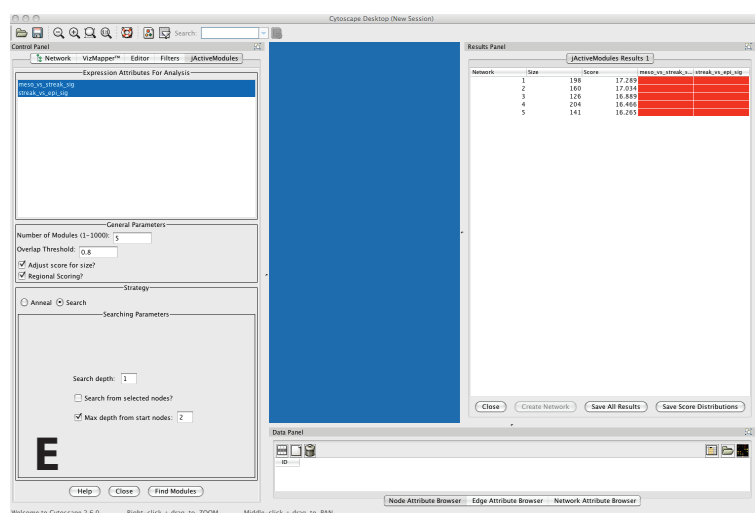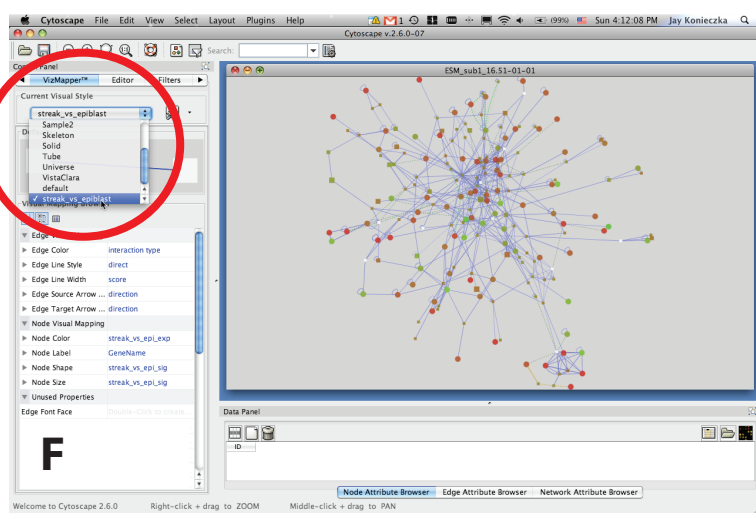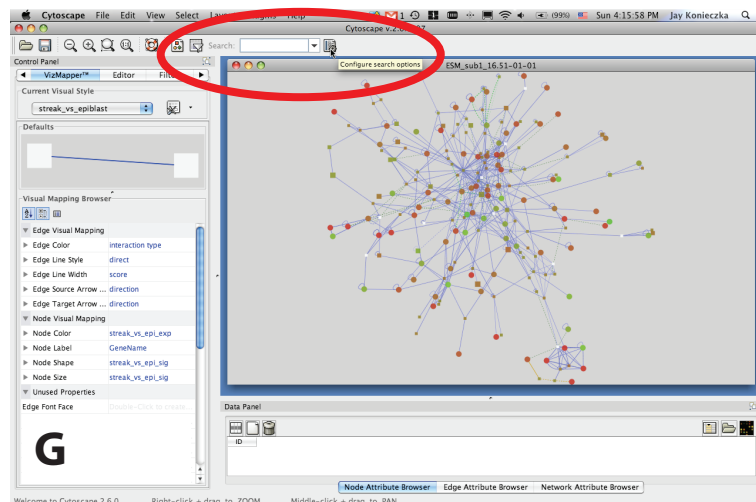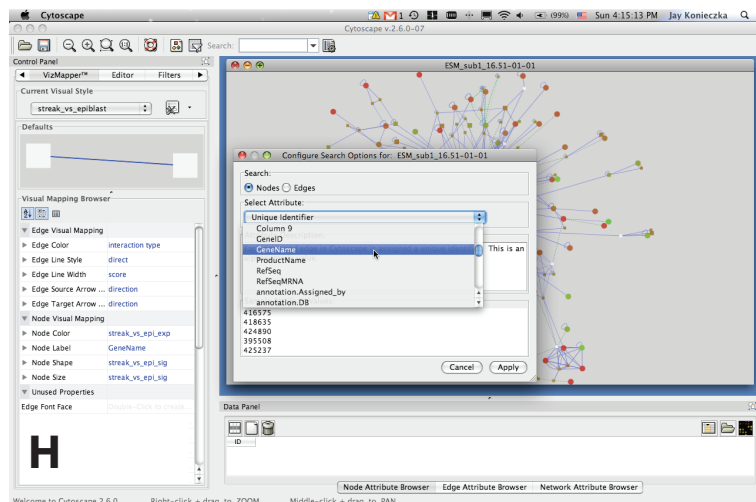

Supplement: Additional file 3 — This file contains screen-shots of each step of part 2 of the main tutorial to help user's follow along. [file 1471-2164-10-S2-S6-S3.pdf]

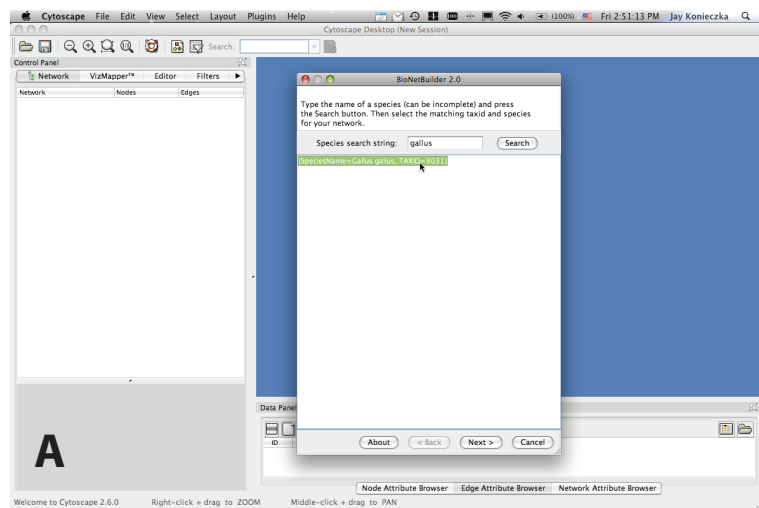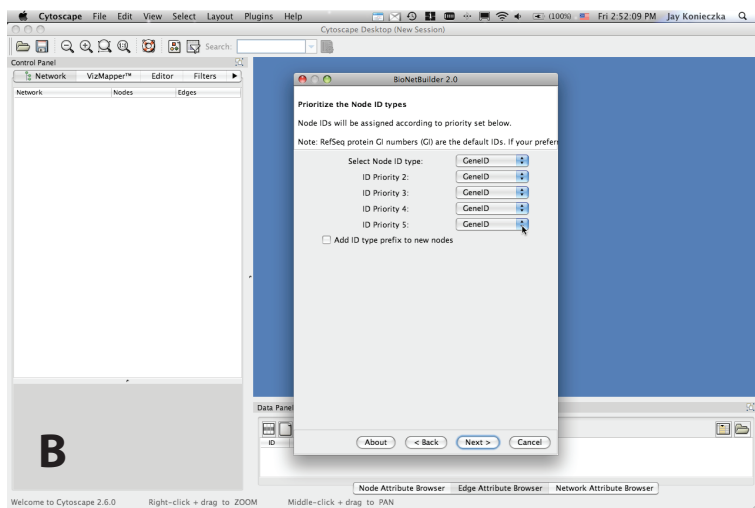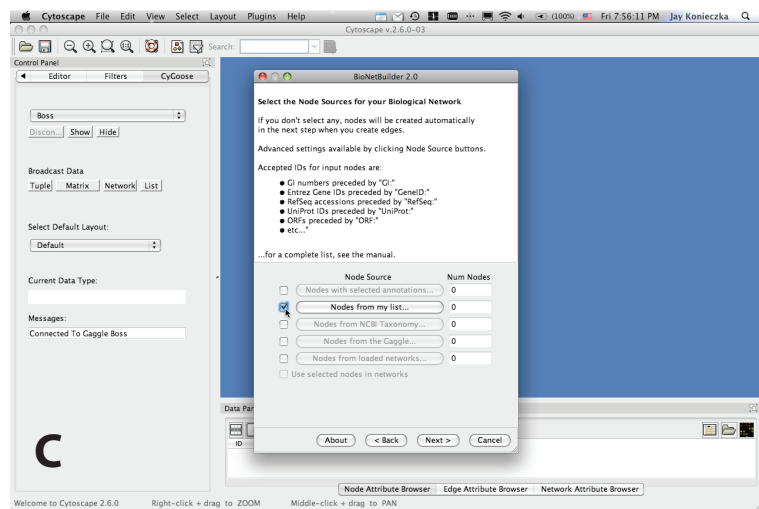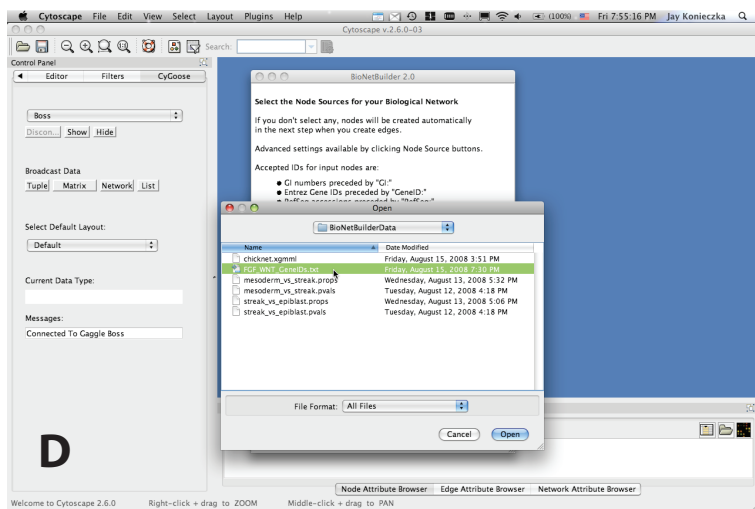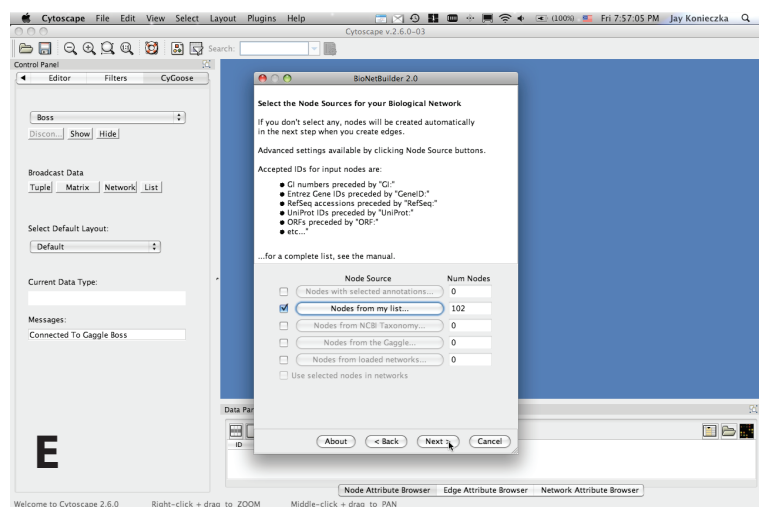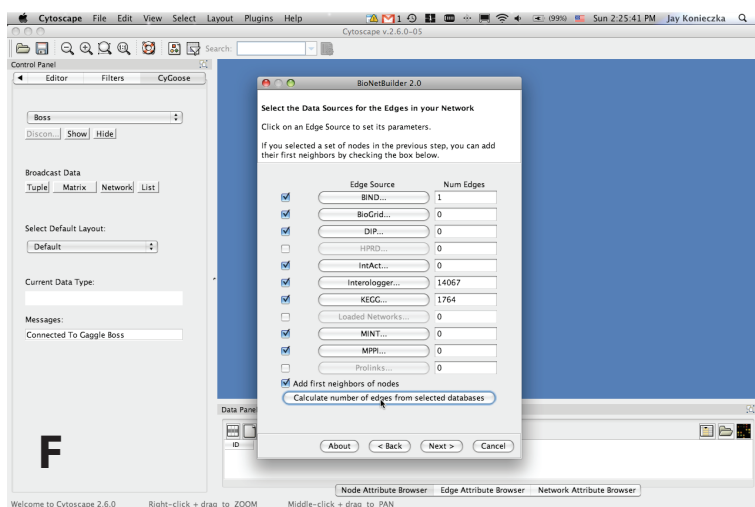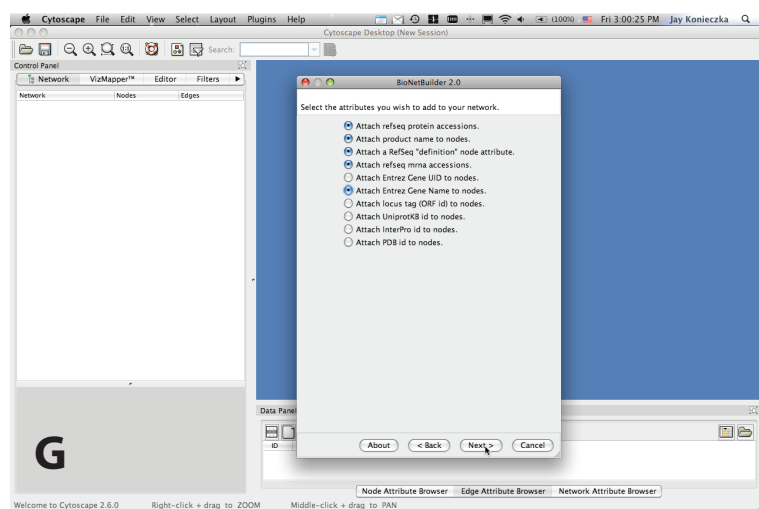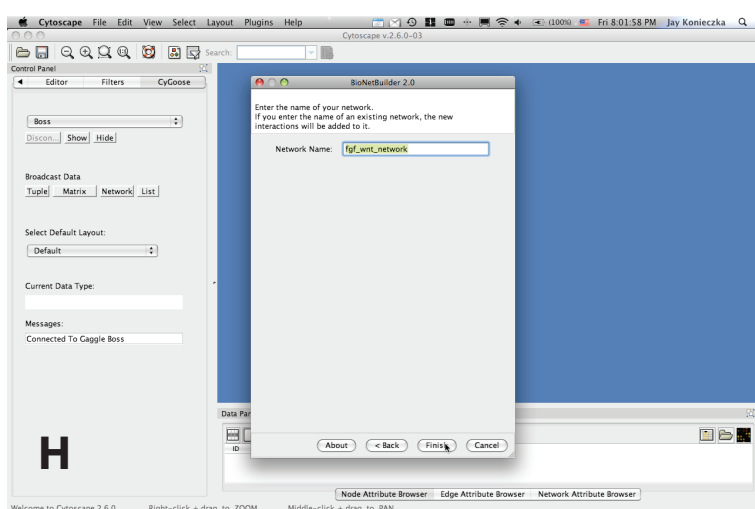

Supplement: Additional file 5 — This file contains screen-shots of each step of the alternate tutorial to help user's follow along. [file 1471-2164-10-S2-S6-S5.pdf]
