# Supplementary material for: BioNetBuilder2.0: bringing systems biology to chicken and other model organisms
Source: BMC Genomics. 2009 Jul 14;10(Suppl 2):S6. doi: 10.1186/1471-2164-10-S2-S6 (PMC2966329; doi:10.1186/1471-2164-10-S2-S6)
Supplement: Additional file 4 — Alternate tutorial. [file 1471-2164-10-S2-S6-S4.docx]

# Alternate Tutorial

### The purpose of this tutorial is to illustrate an additional case where BioNetBuilder can be used to obtain interaction data addressing a biological question. In this case, we are interested in the same question as that in the main text, and we will construct a network starting from a list of relevant genes. Supposing the microarray results alone piqued interest in the FGF and non-canonical WNT pathways, we constructed a list of Entrez Gene IDs representing all members of each of these pathways listed in the KEGG pathways database at <http://www.genome.jp/dbget-bin/get_pathway?org_name=gga&mapno=04010> and <http://www.genome.jp/dbget-bin/www_bget?pathway+gga04310>, respectively. Here, we construct a network from these WNT and FGF pathway genes provided in the file “FGF_WNT_genes.txt” in the supplemental data package:

###

### *If you are continuing on from the tutorial in the main text, proceed immediately to step 3.*

## Under the *Plugin* drop-down menu, you should now have an option to select the *BioNetBuilder Wizard…* button. This launches BioNetBuilder. Type “gallus” in the “Species search string:” text box and click *Search*. After a moment, “SpeciesName=Gallus gallus, TAXID=9031” will be returned in the text area below (Figure S3A); select that line in the text area and click *Next >*.

## You will now see the Node ID type selection pane. You will notice 5 ordered options for selecting Node ID types. These are used for selecting your preference for node identifiers. For this tutorial, we will need to have all of our node identifiers as Entrez Gene UID numbers. Select GeneID for each of the 5 options (Figure S3B), then click *Next >*.

## You will now see a pane for selection of Node Sources for your Biological Network. In this tutorial, we will demonstrate construction of a smaller network constructed from a set of starting nodes, in this case nodes from our non-canonical WNT and FGF list. Click on the checkbox next to “Nodes from my list…” and click *Nodes from my list…* once the button is enabled (Figure S3C). Navigate to “FGF_WNT_GeneIDs.txt” in the downloaded supplemental folder “BioNetBuilderData” (Figure S3D) and click *Open*. You should see “102” in the “Num Nodes” text area (Figure S3E).

## You will now see the pane for selecting “Data Sources for the Edges in your Network” (Figure S3F). Select the boxes next to the following to select the resources that could have interactions available for chicken: *BIND*, *BioGrid*, *DIP*, *IntAct*, *Interologger*, *KEGG*, *MINT*, and *MPPI*. We will also obtain all adjacent interactions to those found in the FGF and non-canonical WNT pathways. Click on the “Add first neighbors of nodes” checkbox. Now click *Calculate number of edges from selected databases* to display the number of interactions available for chicken from each resource. Because few interactions have been verified directly in chicken, almost all of the interactions come from KEGG and Interologger, the latter being those transferred from other species. If you wish to restrict this set based on scores and/or interaction types, you may do so by clicking on the *Interologger…* button and setting your criteria. The confidence threshold is a value (between 0 and 1) above which interolog-derived interactions will be included. The scoring system is explained in detail in Konieczka et al [20]. For this tutorial, leave the threshold set at 0.0 in order to return all available interactions. Click *Next >* to advance to the attributes pane.

## You should now see the pane for selecting “attributes you wish to add to your network” (Figure S3G). Although all node identifiers will be Entrez Gene UIDs due to our selection in step 2, we can attach various other data to our nodes as well by selecting them here. When you are satisfied with your selections, click *Next >* to advance to the Network name panel.

## Finally, you must set your “Network Name” by entering it in the text field (Figure S3H). Click *Finish* to construct your network. Although considerably smaller than the entire network, it is still quite large and can take quite a while to construct. If you wish to skip this download and load the network directly, we have included the file “fgf_wnt_net.xgmml” in the supplemental data package. You can import this network by selecting *File* 🡪 *Import* 🡪 *Network (multiple file types)…* Alternatively, you can back up to step 4 and deselect the “Add first neighbors of nodes” checkbox in order to download a much smaller network more quickly.

Expression data can now be loaded by proceeding to steps 1 and 2 of the “Getting Value from the Chicken Interactome” tutorial in the main text. If desired, jActiveModules can be run on this network according to steps 3 and 4 to obtain the most relevant portions of this FGF/non-canonical WNT subnetwork. It is interesting to note that, in comparison to the subnetwork obtained using jActiveModules on the entire network as detailed in the main tutorial, the subnetwork retrieved here does not contain DLG1 (discs large). This is because DLG1 is not a first neighbor of the genes in the FGF and WNT pathways. This gene’s relevance could only be discovered when including all interactions.
